# Cleaving Ergot Alkaloids by Hydrazinolysis—A Promising Approach for a Sum Parameter Screening Method

**DOI:** 10.3390/toxins13050342

**Published:** 2021-05-11

**Authors:** Maximilian Kuner, Susanne Kühn, Hajo Haase, Klas Meyer, Matthias Koch

**Affiliations:** 1 Bundesanstalt für Materialforschung und-prüfung (BAM), 12205 Berlin, Germany; maximilian.kuner@bam.de (M.K.); klas.meyer@bam.de (K.M.); 2 Institut Kirchhoff Berlin GmbH, 13347 Berlin, Germany; susanne.kuehn@mxns.com; 3 Department of Food Chemistry and Toxicology, Technische Universität Berlin, 10623 Berlin, Germany; haase@tu-berlin.de

**Keywords:** ergot alkaloids, sum parameter method, hydrazinolysis, esterification

## Abstract

Ergot alkaloids are mycotoxins formed by fungi of the *Claviceps* genus, which are some of the most common contaminants of food and feed worldwide. These toxins are a structurally heterogeneous group of compounds, sharing an ergoline backbone. Six structures and their corresponding stereoisomers are typically quantified by either HPLC-FLD or HPLC-MS/MS and the values subsequently summed up to determine the total ergot alkaloid content. For the development of a screening method targeting all ergot alkaloids simultaneously, the alkaloids need to be transferred to one homogeneous structure: a lysergic acid derivative. In this study, two promising cleaving methods—acidic esterification and hydrazinolysis—are compared, using dihydroergocristine as a model compound. While the acidic esterification proved to be unsuitable, due to long reaction times and oxidation sensitivity, hydrazinolysis reached a quantitative yield in 40‒60 min. Parallel workup of several samples is possible. An increasing effect on the reaction rate by the addition of ammonium iodide was demonstrated. Application of hydrazinolysis to a major ergot alkaloid mix solution showed that all ergopeptines were cleaved, but ergometrine/-inine was barely affected. Still, hydrazinolysis is a suitable tool for the development of a sum parameter screening method for ergot alkaloids in food and feed.

## 1. Introduction

Ergot alkaloids are secondary metabolites formed by Claviceps fungi of which *Claviceps purpurea* is the most common species in Europe [1,2]. The most familiar host of these fungi is rye, but wheat, triticale or other grasses may also be infested [2]. During the infestation, sclerotia—the wintering body of the fungus—are formed, which contain the toxic ergot alkaloids in varying concentrations [3]. Claviceps fungi and the corresponding alkaloids have played an important role since the Middle Ages, leading to tens of thousands of deaths [4,5]. When ingested continuously, ergot alkaloids can cause two types of disease: the gangrenous or the convulsive type of ergotism, either one eventually resulting in death [5,6].

**Figure 1:**
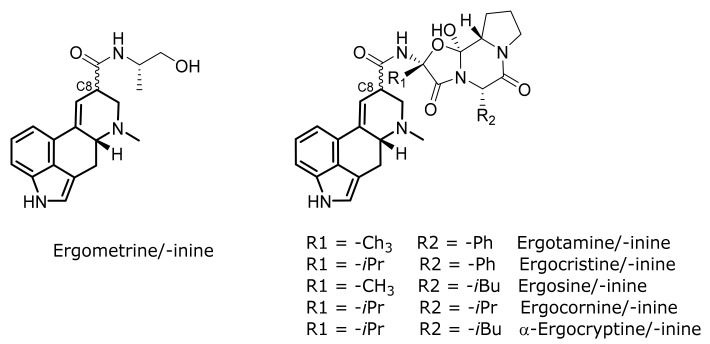
The 12 major ergot alkaloids with highlighted ergoline structure (right: ergopeptines).

More than 50 naturally occurring, plus further synthetic or semisynthetic, compounds belong to the group of ergot alkaloids. Six compounds and their corresponding stereoisomers (Figure 1) are most commonly found in sclerotia [7]. These are typically measured when it comes to ergot alkaloid quantification. All ergot alkaloids share a tetracyclic ring system: the ergoline structure. The major ergot alkaloids just differ in their substituents at the C8 position. Different classes can be distinguished at this substituent: clavine alkaloids, simple lysergic acid derivatives (e.g., ergometrine), ergopeptames and ergopeptines, which contain a cyclic tripeptide moiety (e.g., ergotamine, ergosine, etc.). Isomerization at the chiral C8 position leads to the formation of corresponding stereoisomers. Consequently, both isomers have to be quantified, even though the S-isomers (suffix: -inine, e.g., ergometrinine) are biologically inactive [1,7].

For the analysis of ergot alkaloids in food/feed samples, numerous methods have been described, most of them quantifying each of the 12 major ergot alkaloids separately. Typically, HPLC with either fluorescence or MS/MS detection is used for quantification [1,8,9,10]. However, for the safety assessment of food samples, the fraction of each single compound to the total amount of ergot alkaloids is irrelevant, as only the sum is used for the evaluation. To date, just a few methods have been published for measuring the total amount of ergot alkaloids without quantifying each of the major ergot alkaloids individually [10,11,12,13].

The oldest method used to determine the sum of ergot alkaloids is the van Urk reaction, forming a purple compound, which is quantified colorimetrically [12]. This method is very unspecific, as the reagents used react with all indole-containing compounds in the sample, e.g., tryptophan [14]. Another quick sum parameter method is the enzyme-linked immunosorbent assay (ELISA). Due to the structural diversity of ergot alkaloids, the development of suitable antibodies to bind all ergot compounds specifically is challenging. Some ELISA kits are commercially available, but comparative studies showed poor consistency of the results obtained by ELISA when being compared to the ergot levels measured by high-performance liquid chromatography–fluorescence detection/mass spectrometric detection (HPLC-FLD/MS-MS) [15,16,17,18]. All these described sum parameter methods target the ergot alkaloids directly and thus suffer from cross-reactivity caused either by structural diversity of the ergots or an unspecific reaction (van Urk).

An approach published by Oellig et al. is to transfer the ergot alkaloids to one uniform structure [11]. Oellig et al. applied a mixture of lithium triethylborohydride and methanol to a toluene sclerotia extract. Transformation of all ergopeptines to lysergic acid amide was observed, while ergometrine/-inine remained unaffected. Subsequently, lysergic acid amide was quantified by high-performance thin layer chromatography (HPTLC-FLD). The obtained values were in good accordance with the HPLC-FLD reference data of the unreacted sample [11].

Transformation of all ergot alkaloids to one uniform compound would have several advantages. Fewer calibration standards are needed, resulting in lower costs, which is of particular interest for routine analysis. Expected shorter HPLC runtimes lead to higher throughput and thus lower the analysis costs, too. A simplification of the HPLC spectrum analysis is also expected because fewer peaks need to be integrated.

Suitable target structures are lysergic acid derivatives, as all ergot compounds share the ergoline moiety [7]. The stability of amide groups hinders the reaction, but some methods are still described in the literature attacking this bond [19]. Alkaline or acidic hydrolysis leads to the formation of either lysergic acid or lysergic acid amide, depending on the harshness of the conditions used [19,20,21]. The formation of the corresponding lysergic acid esters in acidic alcoholic solutions is also described in a patent [22,23]. The amide bond can also be broken with reducing agents, as described by Stoll et al. using lithium aluminum hydride (LiAlH_4_), leading to three different products [24]. Another reaction targeting the amide bond of the ergot alkaloids is hydrazinolysis, leading to the formation of lysergic acid hydrazide [25]. Due to the good crystallization properties of the reaction product, this method can be used for the synthesis of ergot alkaloid-based drugs even with low-concentrated ergot alkaloid solutions as a starting material [26].

**Figure 2:**
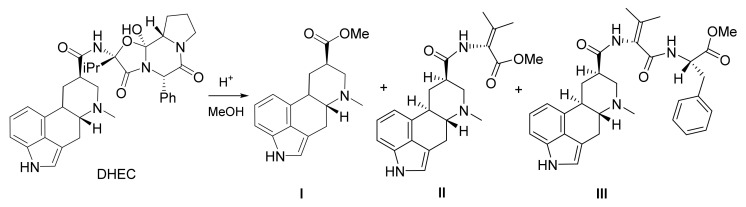
Acidic esterification of dihydroergocristine (DHEC). Main product: dihydrolysergic acid methylester (**I**) and main partially cleaved byproducts (**II** and **III**).

In brief: a sum parameter method would be highly advantageous compared to the classical HPLC-FLD or HPLC-MS/MS approach. Owing to the structural diversity of the ergot alkaloids, cleavage to a simple lysergic acid derivative as a common feature in all ergot compounds is indispensable. Some methods for the reaction of the amide bond in ergot alkaloids are described in the literature. The aim of the present study is the optimization of two ergot alkaloid cleaving methods: acidic esterification and hydrazinolysis. Furthermore, both methods should be compared not just by means of reaction yields, but also regarding other aspects for a possible routine screening method, like handling of the reaction or the possibility of parallelization. Inexpensive, easy to handle reagents and the formation of just a few defined products lead to the choice of these two reactions. The reaction conditions should be enhanced by means of reaction temperature, the addition of catalysts or microwave assistance. For the optimization experiments, dihydroergocristine (DHEC) (Figure 2) was used as a model compound, due to better availability than the native ergot alkaloids. Another advantage of DHEC is the suppression of isomerization at the C8 position during the reaction, leading to better analyzable reaction mixtures.

## 2. Results

### 2.1. Acidic Esterification

**Figure 3:**
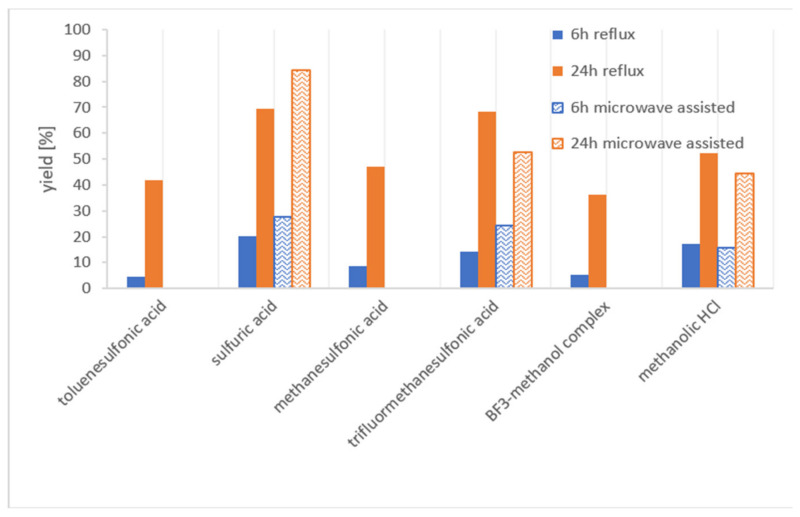
Yields of **I** determined by HPLC-UV (210 nm). Reaction conditions: DHEC and the corresponding acid were dissolved in methanol under inert atmosphere and refluxed (76 °C oil bath) or microwaved (set to 76 °C in temperature control mode). Samples were taken after 6 h and 24 h.

The acidic esterification was conducted by dissolving the corresponding acid and DHEC in methanol under nitrogen atmosphere and subsequently refluxing the solution. HPLC-MS of the reaction mixture showed, next to the desired dihydrolysergic acid methylester **I**, several partially cleaved byproducts **II** and **III** (Figure 2), whose structures were elucidated by high-resolution MS/MS experiments. Due to the side products, reaction yield determination of **I** by measuring the decrease in the DHEC concentration in the reaction mixture by HPLC-UV was impossible. NMR also proved to be unsuitable, owing to the complex reaction mixture. Hence, **I** was synthesized and purified to be used as a calibration standard in an HPLC-UV method. For the synthesis, the reaction time was prolonged to five days, so that all partially cleaved side products were transformed to **I**. The developed HPLC-UV method was used to determine the reaction yields of the acidic esterification regarding **I**, using varying reagents (Figure 3).

Maximum yields of about 20% were observed after 6 h and around 70% after 24 h. To increase the reaction yields, the three most promising reagents were used in a microwave-assisted approach. Compared to the previously used Schlenk flasks, keeping an inert atmosphere in the microwave reaction vessels was challenging. Thus, the heavier argon was used instead of nitrogen to prevent oxygen contamination. Despite this, the reaction had to be repeated several times, due to oxidation. The contamination of the reaction with oxygen is easily visible by an intense purple coloring of the reaction mixture. A literature search showed that ergolines can be oxidized in acidic media to a dimer showing an intense purple color at low pH [27]. An increase in the yield of **I**, when using microwave assistance, was observed for the reagents sulfuric and trifluormethane sulfonic acid after 6h. The maximum yield was 84% after 24 h using sulfuric acid.

### 2.2. Hydrazinolysis

**Figure 4:**
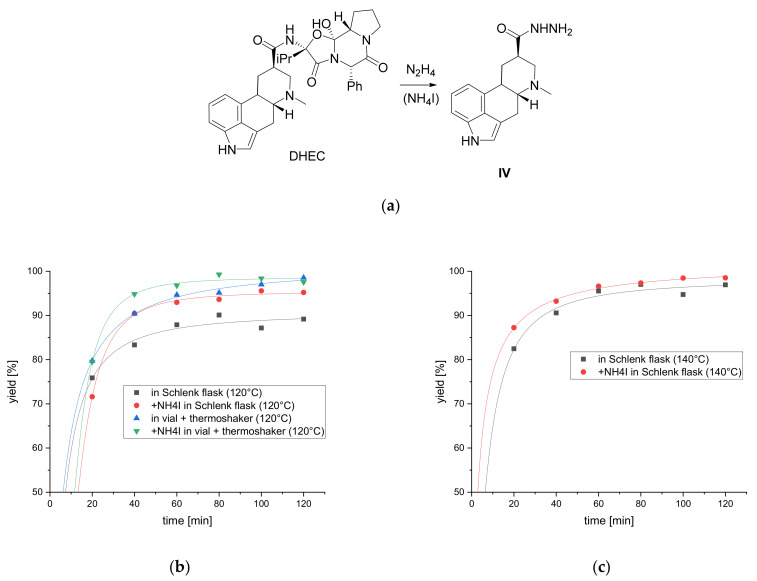
Hydrazinolysis of DHEC to **IV** with hydrazine hydrate. (**a**) Reaction scheme of the hydrazinolysis. To some reaction mixtures, NH_4_I was added to promote the reaction as described by Shimizu et al. [28] (**b**) Yields of **IV** measured by HPLC-UV (254 nm) against reaction time at 120 °C. The reaction was conducted either in a Schlenk flask heated in an oil bath or in headspace vials heated and stirred in a thermoshaker. (**c**) Yields of **IV** measured by HPLC-UV (254 nm) against reaction time at 140 °C. The reaction was conducted in a Schlenk flask heated in an oil bath.

Shimizu et al. described ammonium salts as potent accelerating reagents for the hydrazinolysis of various amides. In their study, ammonium iodide was found to be the best compound to promote the reaction [28]. Thus, the impact of ammonium iodide on the hydrazinolysis was tested. To determine the reaction yield of the hydrazinolysis, dihydrolysergic acid hydrazide **IV** was synthesized (Figure 4a) and purified. The purity of **IV** was determined by quantitative NMR (q-NMR). **IV** was used as a calibration standard and an HPLC-UV method was developed to determine the reaction yield.

For the hydrazinolysis study, DHEC was suspended in hydrazine hydrate (65%) under nitrogen atmosphere. The suspension was heated, and a clear solution was obtained during the reaction. To see whether ammonium salts accelerate the reaction, one equivalent ammonium iodide was added to the reaction mixture. Additionally, different temperature levels (100 °C, 120 °C, 140 °C) were tested to optimize the reaction conditions. Samples were taken every 20 min for 2h and measured with HPLC-UV. After the first satisfactory results, the reaction vessels were changed from Schlenk flasks in an oil bath to headspace vials, which were heated and stirred in a thermoshaker. This has two advantages: the complexity of the experimental setup is reduced and thus several reactions can be performed in parallel. Headspace vials were chosen due to their good pressure resistance. The reaction yields were plotted against the reaction time (Figure 4b,c) (curves for 100 °C in the Supplementary Materials).

As expected, the yields of **IV** improved with increasing reaction temperature. Moreover, the addition of ammonium iodide improved the reaction rate, leading to an increased yield of about 5% after 40 min. Further enhancement was observed, when conducting the reaction in vials heated in a thermoshaker. Better mixing of the reaction components, when shaking the vials instead of stirring with a magnetic stirrer, led to an increased yield and shorter reaction times till quantitative reaction. Due to leakage in the septum above the boiling point of the reaction mixture, the reaction could not be properly conducted in vials at 140 °C. Therefore, these values are not included in Figure 4c.

As the results of the hydrazinolysis were quite satisfactory, hydrazinolysis was finally applied to a mix of the 12 major ergot alkaloids (see Figure 1). After 1h, all ergopeptine signals were untraceable in HPLC-FLD and HPLC-MS, while the signals of ergometrine/-inine remained nearly unchanged. Two isomeric forms of lysergic acid hydrazide were confirmed as reaction products by HPLC-MS (chromatograms in the Supplementary Materials), indicating successful cleavage of the native ergopeptines. A potential explanation for the inertness of ergometrine/-inine might be the missing neighboring effect in ergometrine, which is described in the literature for peptides with at least two vicinal amide bonds. As ergometrine contains just one amide bond, destabilization by adjacent amide moieties does not occur [29].

## 3. Discussion

All in all, the yields of the acidic esterification are too low after 6 h. The maximum achieved yield of 84% after 24 h would be sufficient for a screening method, but 24 h is an unacceptably long reaction time for a quick sum parameter method. With increasing pK_a_ (increasing strength of the acid), an increased reaction rate was observed. Enhancement could also be achieved by using microwave assistance. The biggest disadvantage of the method is the susceptibility to oxidation, which requires Schlenk flasks and nitrogen atmosphere. Thus, parallel workup of several samples is possible to a limited degree. Overall, the acidic esterification is inappropriate for use in a screening method.

The yields achieved by hydrazinolysis were satisfactory. A 95% yield of **IV** after 40 min and quantitative yield after 1 h are sufficiently quick for a sum parameter method. Adding ammonium iodide had an increasing effect on the reaction rate. Parallel cleaving of several samples is possible, as the reaction can be conducted in vials. This is also advantageous, as a further increase in the reaction yield was observed, due to the more thorough mixing of the reaction compounds in the thermoshaker. Optimum cleavage conditions for a possible sum parameter method are: 120 °C, reaction mixed and heated in a thermoshaker under inert atmosphere for 40 min in a vial. Flushing the vial with inert gas prior to hydrazinolysis is sufficient and easily practicable.

Compared to the previously published approach of Oellig et al. [11], this method is faster (2 h vs. 40 min) and involves fewer problematic reagents (hydrazine hydrate instead of lithium triethylborohydride, reacting violently with water or alcohols). Hydrazinolysis as well as the reductive approach suffer from not targeting ergometrine/-inine.

However, the hydrazinolysis cleavage reaction can be considered as a suitable approach for the development of a screening method for the ergot alkaloid content as a sum parameter. In addition, an automation of the screening method should also be feasible.

## 4. Materials and Methods

### 4.1. Chemicals and Equipment

All chemicals were used as purchased without further purification. DHEC mesylate was purchased from Teva Czech Industries s.r.o. (Prague, Czech Republic). All native ergot alkaloids were obtained from RomerLabs Division Holding GmbH (Tulln, Austria). Hydrazine hydrate, methane sulfonic acid, boron trifluoride methanol solution, tetrachloro nitrobenzene and ammonium iodide were obtained from Sigma-Aldrich (St. Louis, MO, USA). Dry methanol was purchased from Acros Organics (Ghent, Belgium). DMSO, *iso*-propanol, dichloromethane (DCM) and acetonitrile (MS grade) were obtained from Th. Geyer (Renningen, Germany). Ammonium acetate was obtained from J.T. Baker (Deventer, Netherlands). Sulfuric acid was purchased from Merck KgaA (Darmstadt, Germany). Methanolic hydrochloric acid was obtained from Bernd Kraft GmbH (Duisburg, Germany). Trifluormethane sulfonic acid was purchased from ABCR GmbH & Co KG (Karlsruhe, Germany).

Microwave-assisted reactions were conducted in an MLS 1200 Mega system (Milestone, Sorisole, Italy). For thermoshaking, an MHR-13 (HLC, Pforzheim, Germany) was used. Compounds were freeze-dried in a Gamma 1-16 LSCplus (Christ, Osterode, Germany) freeze-drying system.

HPLC-UV/-FLD/-MS were carried out with a 1290 Infinity HPLC system (Agilent, Waldbronn, Germany) coupled to a 6130 quadrupole MS (Agilent, Waldbronn, Germany). For the measurements, a Phenomenex Luna Phenyl Hexyl column (250 × 4.6 mm, 5 µm) was used.

Preparative HPLC was performed on a 1260 preparative system (Agilent, Waldbronn, Germany) coupled to a 6130 quadrupole MS (Agilent, Waldbronn, Germany). A Phenomenex Luna Phenyl Hexyl column (250 × 21.2 mm, 100 µm) was used.

High accurate masses were measured with a TripleTOF 6600 mass spectrometer (Sciex, Darmstadt, Germany) coupled to a 1290 Infinity II system (Agilent, Waldbronn, Germany). For the measurements, an Agilent Zorbax Eclipse Plus C18 column (50 × 2.1 mm, 1.8 µm) was used.

^1^H-NMR-spectra were measured on a MercuryPlus 400 (Varian, Palo Alto, California USA) spectrometer at 400 MHz, ^13^C-NMR at 100 MHz.

^1^H quantitative NMR spectra (q-NMR) were measured on a VNMRS (Varian, Palo Alto, OH, USA) spectrometer at 500 MHz. An XP2 U/M (Mettler Toledo, Columbus, OH, USA) ultra-micro balance was used to weigh the sample and the standard (1,2,4,5-Tetrachloro-3-nitro-benzene, purity: 99.86%, *Trace*-Cert©, Sigma-Aldrich). Samples were dissolved in DMSO-d_6_.

All samples and calibration curves were produced and evaluated under gravimetric control.

### 4.2. Synthesis of Dihydrolysergic Acid Methylester (I)

To a stirred solution of DHEC mesylate (1.522 g, 2.15 mmol) in dry methanol (50 mL), concentrated sulfuric acid (4.7 mL, 88 mmol, 41 eq) was added under nitrogen atmosphere in a Schlenk flask. The reaction mixture was heated to 55 °C and stirred for 5 days. The reaction mixture was poured into aqueous ammonia solution (50 mL 25% ammonia solution +50 mL water) and extracted with DCM (4 × 50 mL). Organic phases were collected, and all solvents removed by rotary evaporation. Clean up was conducted via preparative HPLC (acetonitrile: 0.02% aqueous NH_4_Ac, 40:60, 20 min runtime). Fractions containing the target substance were combined, most of the solvent removed by rotary evaporation and the aqueous residue extracted with DCM (3 × 20 mL). The organic phase was dried with MgSO_4_ and removed by rotary evaporation. Dihydrolysergic acid methylester (0.198 g, 0.69 mmol, 32%) was obtained as a white solid. Purity of the compound determined by q-NMR was 86.2%.

**m/z (measured) (M+H)^+^** = 285,1608 (theoretical **(M + H)^+^**: 285,1598, δ = 3.5 ppm).

**^1^H-NMR (400 MHz, CDCl_3_):** δ (ppm) **=** 8.04 (s, 1H, N*H*), 7.19 (d, *J* = 1.6 Hz, 1H, C*H*_ar_), 7.16 (d, *J* = 8.2 Hz, 1H, C*H*_ar_), 6.95 (dt, *J* = 6.3, 1.4 Hz, 1H, C*H*_ar_), 6.89 (t, *J* = 1.9 Hz, 1H, C*H*_ar_), 3.76 (s, 3H, OC*H*_3_), 3.42 (dd, *J* = 14.7, 4.3 Hz, 1H, C*H*), 3.35 – 3.26 (m, 1H, C*H_2_*), 3.11 – 2.95 (m, 3H, C*H*), 2.75 (t, *J* = 13.0 Hz, 1H, C*H_2_*), 2.54 (s, 3H, C*H*_3_), 2.41 (t, *J* = 11.7 Hz, 1H, C*H_2_*), 2.31 – 2.14 (m, 1H, C*H_2_*), 1.71 – 1.49 (m, 1H, C*H_2_*).

**^13^C-NMR (101 MHz, CDCl_3_):** δ (ppm) = 174.14, 133.34, 129.10, 128.55, 126.05, 123.14, 117.82, 113.29, 108.73, 66.74, 58.54, 51.78, 42.87, 41.28, 39.97, 30.49, 26.72.

### 4.3. Yield Determination of the Acidic Esterification

In a Schlenk flask, DHEC mesylate (78 mg, 0.11 mmol) was dissolved in dry methanol (5 mL) under nitrogen atmosphere and the corresponding acid (~55 eq) added. Due to the preset concentration of the methanolic hydrochloric acid, 200 eq of HCl were used, to keep the amount of DHEC and methanol constant compared to the other reactions. The solution was heated to 76 °C (oil bath temperature) and stirred for 24h. After 6h and 24h, samples were taken (~0.1 mL) and diluted with methanol (1.5 mL).

Microwave-assisted reactions were conducted using the same amounts of reagents, under argon atmosphere in microwave reaction vessels. The microwave was run in temperature control mode at 76 °C.

The obtained samples were measured with HPLC-UV (210 nm, acetonitrile: 0.02% aqueous NH_4_Ac, 50:50, 0.8 mL/min, 30 min runtime). A five-point calibration (R^2^: 99.7%) with concentrations of **I** in methanol ranging from 0.040 mg/g to 0.885 mg/g was prepared and used to determine the yield of **I** in the samples.

### 4.4. Synthesis of Dihydrolysergic Acid Hydrazide (IV)

Under nitrogen atmosphere in a Schlenk flask, DHEC mesylate (0.892 g, 1.26 mmol) was added to stirred hydrazine hydrate (13 mL, 263.65 mmol, 209 eq). The white suspension was heated to 140 °C for 24 h. The reaction mixture was stored in the refrigerator until a white solid precipitated. The precipitate was separated via centrifugation and the solid washed with water. Purification was conducted via preparative HPLC (acetonitrile: 0.005% aqueous NH_4_Ac, 30:70, 20 min runtime). After combining all product-containing fractions, removal of the solvents by rotary evaporation and subsequent freeze-drying, the product (0.163 g, 0.57 mmol, 45%) was obtained as a white solid. Purity of the compound determined by q-NMR was 74.3%.

**m/z (measured) (M+H)^+^** = 285,1710 (theoretical **(M+H)^+^**: 285,1710, δ = 0.0 ppm).

**^1^H-NMR (400 MHz, DMSO-d_6_):** δ (ppm) **=** 10.61 (s, 1H, N*H*), 9.10 (s, 1H, N*H*), 7.33 – 7.24 (m, 1H, C*H*_ar_), 7.22 – 7.09 (m, 2H, C*H*_ar_), 6.93 (dt, *J* = 7.2, 0.9 Hz, 1H, C*H*_ar_), 4.67 (s, 2H, N*H*_2_), 3.45 (dd, *J* = 14.7, 4.3 Hz, 1H, C*H_2_*), 3.07 (ddd, *J* = 11.2, 3.8, 1.9 Hz, 1H, CH_2_), 2.99 – 2.87 (m, 1H, C*H*), 2.83 – 2.60 (m, 2H, C*H*_2_), 2.44 (s, 3H, CH_3_), 2.39 (t, *J* = 11.3 Hz, 1H, C*H_2_*), 2.15 (ddd, *J* = 11.0, 9.5, 4.3 Hz, 1H, C*H*), 2.00 (s, 1 H, CH), 1.62 (q, *J* = 12.9 Hz, 1H, C*H_2_*).

**^13^C-NMR (101 MHz, DMSO-d_6_):** δ (ppm) = 172.66, 133.14, 132.36, 125.88, 122.02, 118.48, 111.92, 110.04, 108.70, 66.59, 59.29, 42.63, 40.44, 39.54, 30.73, 26.57.

### 4.5. Yield Determination of the Hydrazinolysis

In a Schlenk flask, DHEC mesylate (0.265 g, 0.378 mmol) was added to hydrazine hydrate solution (5 mL, 105 mmol, 280 eq) under nitrogen atmosphere. For the reactions with ammonium iodide, one equivalent (0.055 g, 0.378 mmol) was added. The suspension was heated to 140 °C, 120 °C or 100 °C. A clear solution was obtained during the reaction. The reaction mixture was stirred for 2h. Samples were taken every 20 min (~0.1 mL) and diluted with DMSO (~1.5 mL).

For the hydrazinolysis in vials, the same amounts were used as in the Schlenk flasks. The 20 mL headspace vials were flushed with nitrogen and sealed tightly with a septum cap after filling them with the corresponding substances. The vials were shaken in the thermoshaker for 2 h and samples taken every 20 min.

The obtained samples were measured with HPLC-UV (254 nm, acetonitrile: 0.02% aqueous NH_4_Ac, 50:50, 0.8 mL/min, 25 min runtime). A five-point calibration (R^2^: 99.9%) with concentrations of **IV** in DMSO ranging from 0.074 mg/g to 1.459 mg/g was used to quantify the yield of **IV**.

### 4.6. Cleavage of the Native Ergot Alkaloids

Of 1 mL ergot alkaloid standard mix solution (concentration: 0.5 ng/g for each major ergot alkaloid) in a vial, all solvents were removed to dryness in a nitrogen stream at 40°C. After addition of 1 mL hydrazine hydrate, the vial was shaken in a thermoshaker for 1h at 120 °C. All solvents were removed in a nitrogen stream (40 °C), the sample redissolved in *iso*-propanol and measured with HPLC-FLD/-MS (HPLC conditions: acetonitrile: 0.02% aqueous NH_4_Ac, 50:50, 0.8 mL/min, 60 min runtime; FLD conditions: excitation: 330 nm, emission: 415 nm; MS conditions: ESI-pos, SIM mode, [M + H]^+^ of major ergot alkaloids and lysergic acid hydrazide).

## Data Availability

Not applicable.

## Supplementary Materials

The following are available online at https://www.mdpi.com/article/10.3390/toxins13050342/s1: Figures S1 and S2: ^1^H and ^13^C-NMR spectrum of **I**. Figure S3: exemplary calibration curve for the yield determination of **I**. Figures S4 and S5: ^1^H and ^13^C-NMR spectrum of **IV**. Figure S6: Exemplary calibration curve for the yield determination of **IV**. Figure S7: Yield of **IV** obtained by hydrazinolysis at 100 °C. Figure S8: HPLC-FLD and HPLC-XIC of ergot alkaloid standard mixture before and after (Figure S9) hydrazinolysis.
