# Supplementary material for: Cleaving Ergot Alkaloids by Hydrazinolysis—A Promising Approach for a Sum Parameter Screening Method
Source: Toxins (Basel). 2021 May 11;13(5):342. doi: 10.3390/toxins13050342 (PMC8151494; doi:10.3390/toxins13050342)
Supplement: Supplementary file 1 [file toxins-13-00342-s001.zip › toxins supplementary template NEW.pdf]

# Supplementary Materials: Cleaving Ergot Alkaloids by Hydrazinolysis—a Promising Approach for a Sum Parameter Screening Method

**Maximilian Kuner, Susanne Kühn, Hajo Haase, Klas Meyer and Matthias Koch**

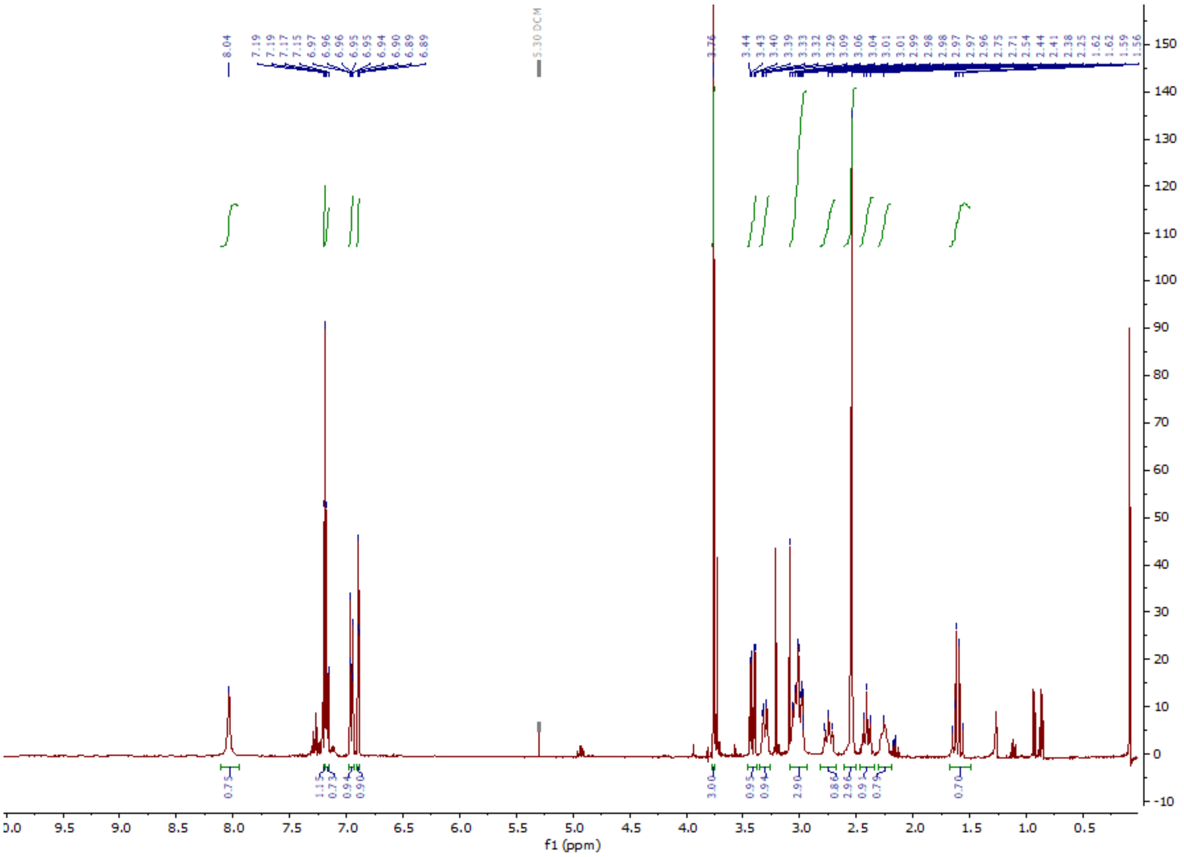


**Figure S1.** $^1$H-NMR of dihydrolysergic acid methylester (I).

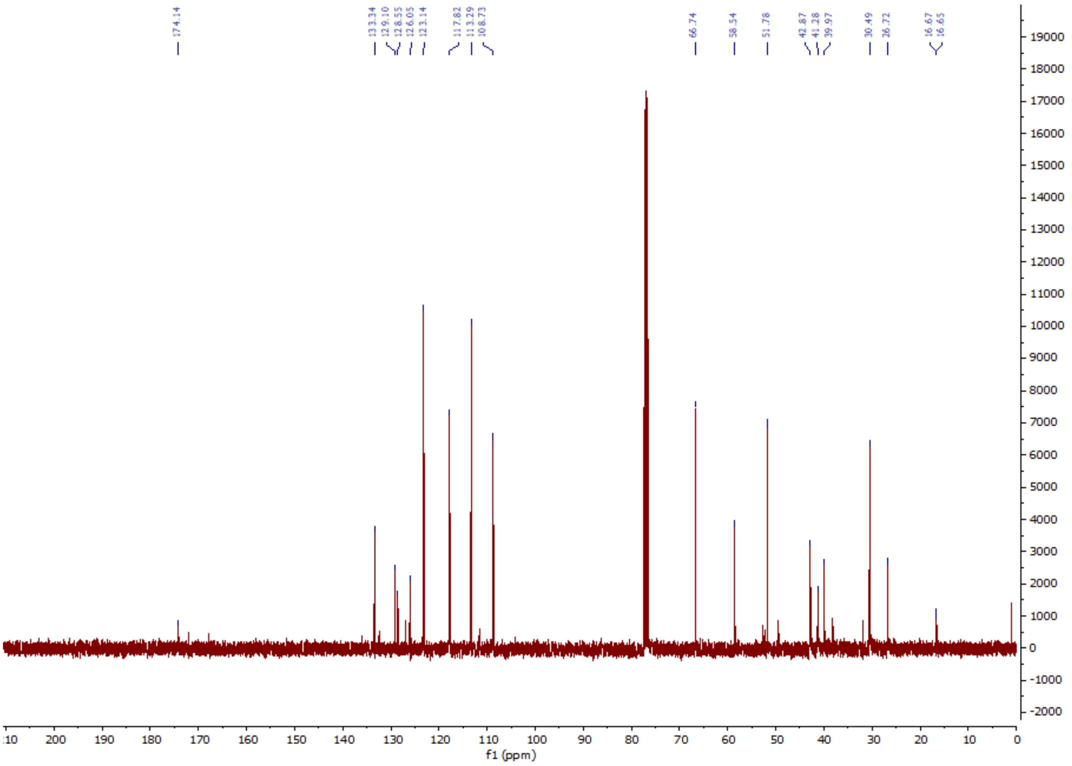


**Figure S2.** $^{13}$C-NMR of dihydrolysergic acid methylester (I).

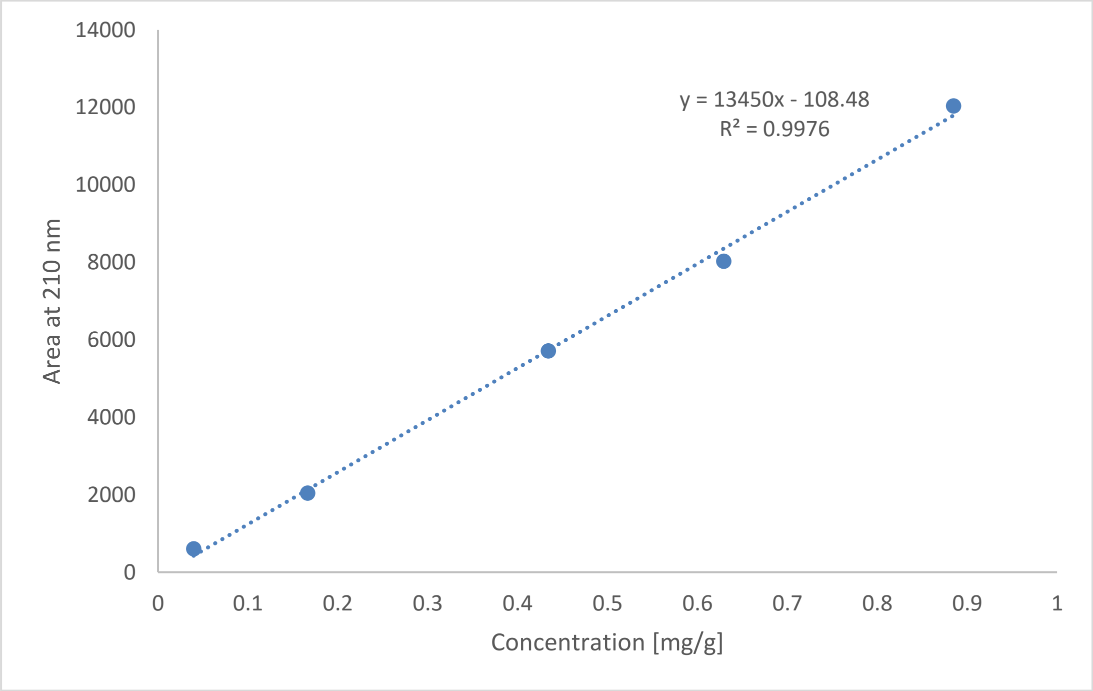


**Figure S3.** Exemplary calibration curve for the yield determination of **I**.

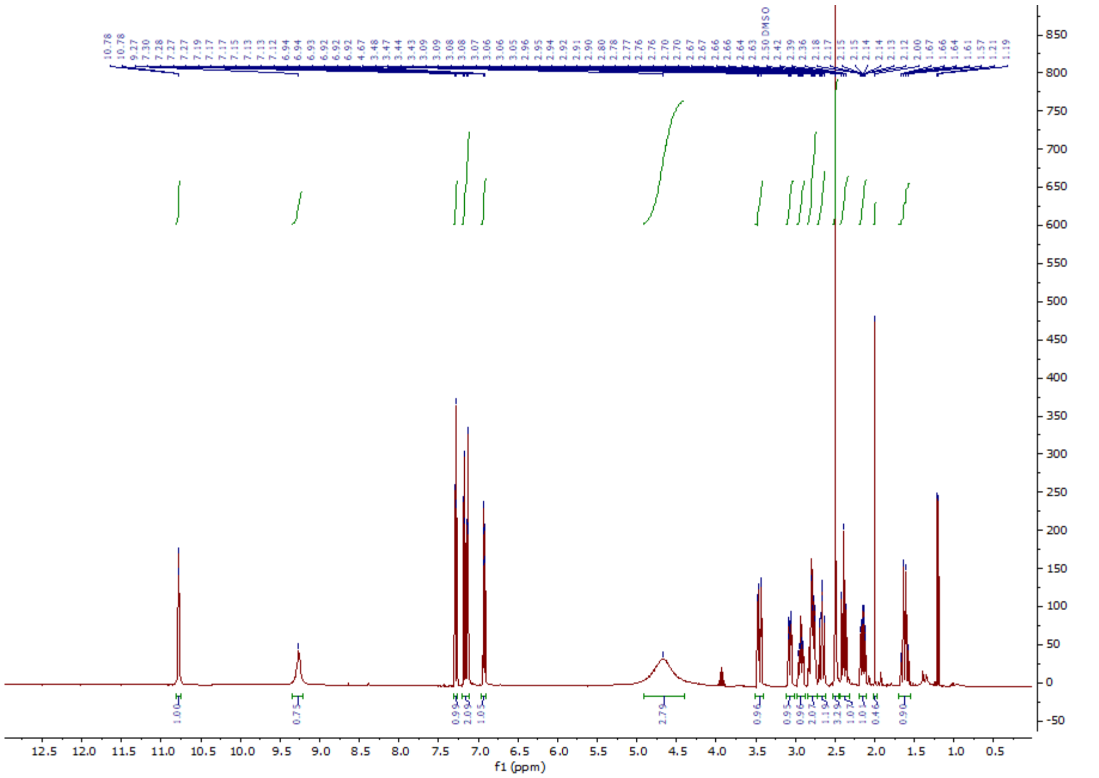


**Figure S4.** $^{1}$H-NMR of dihydrolysergic acid hydrazide (**IV**).

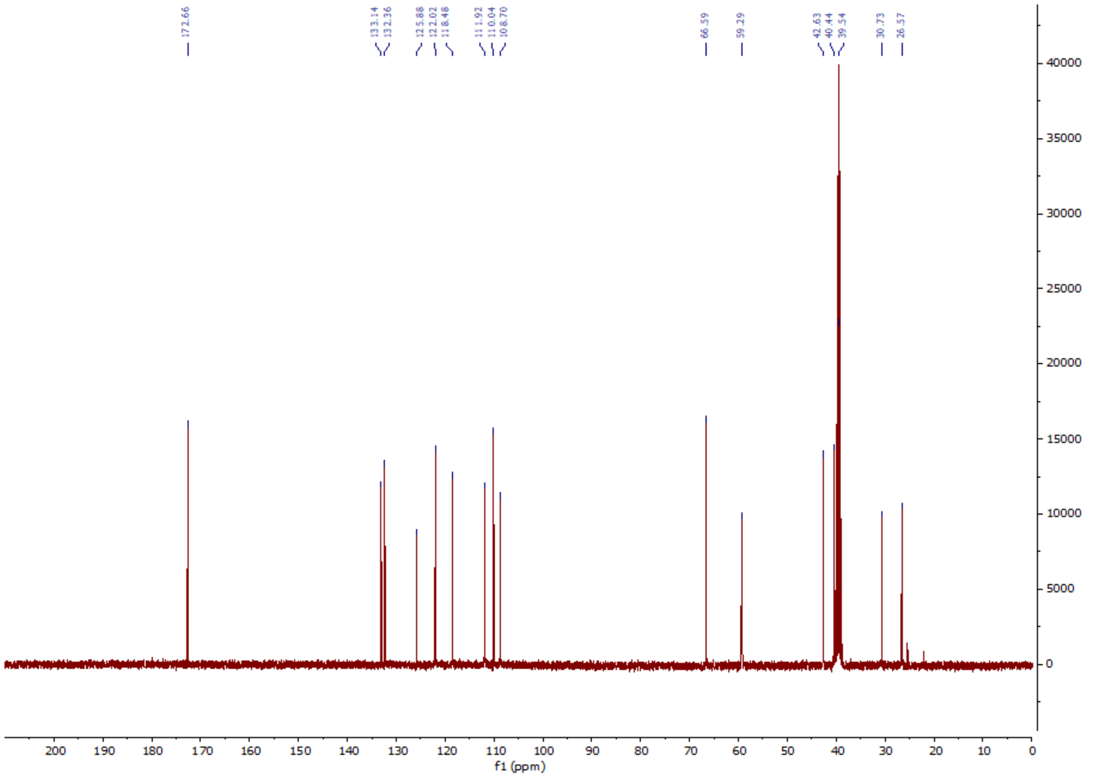


**Figure S5.** $^{13}$C-NMR of dihydrolysergic acid methylester (**IV**).

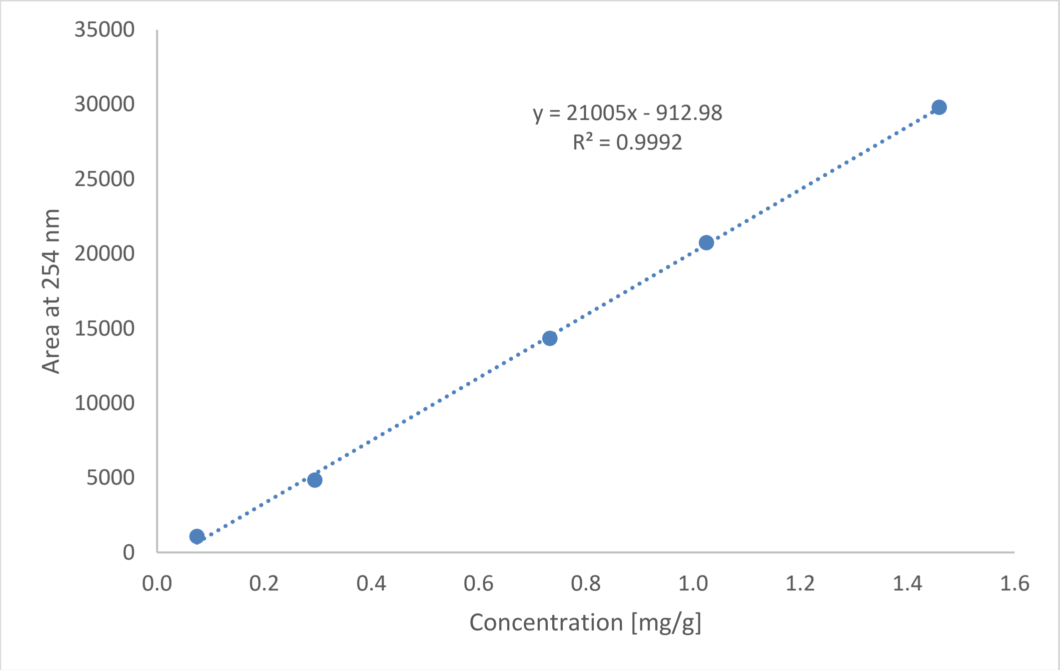


**Figure S6.** Exemplary calibration curve for the yield determination of **IV.**

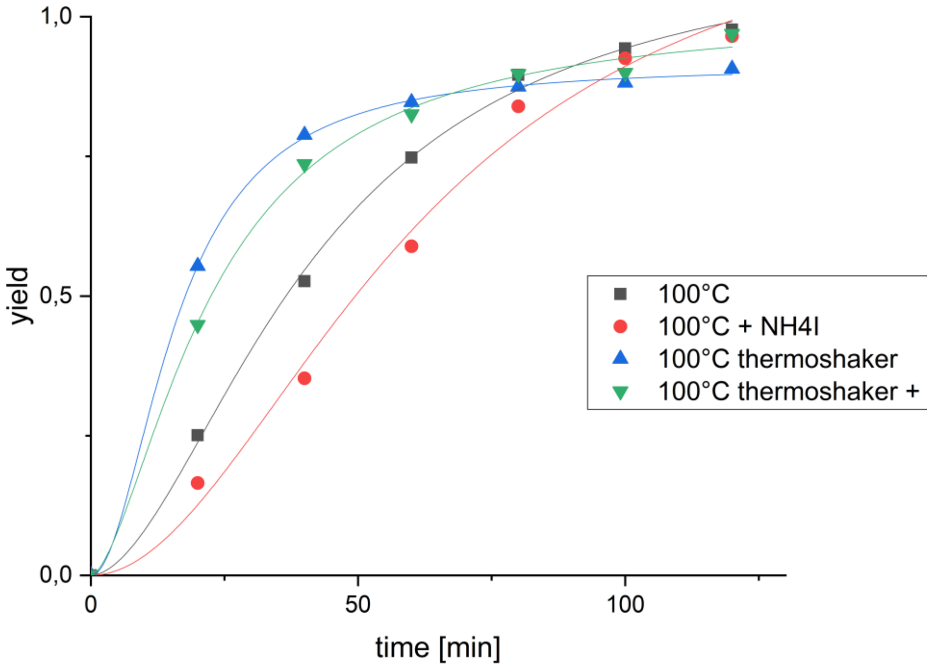


**Figure S7**: Yields of IV measured by HPLC-UV (254 nm) against reaction time at 100°C. The reaction was conducted either in a Schlenk flask heated in an oil bath or in headspace vials heated and stirred in a thermoshaker.

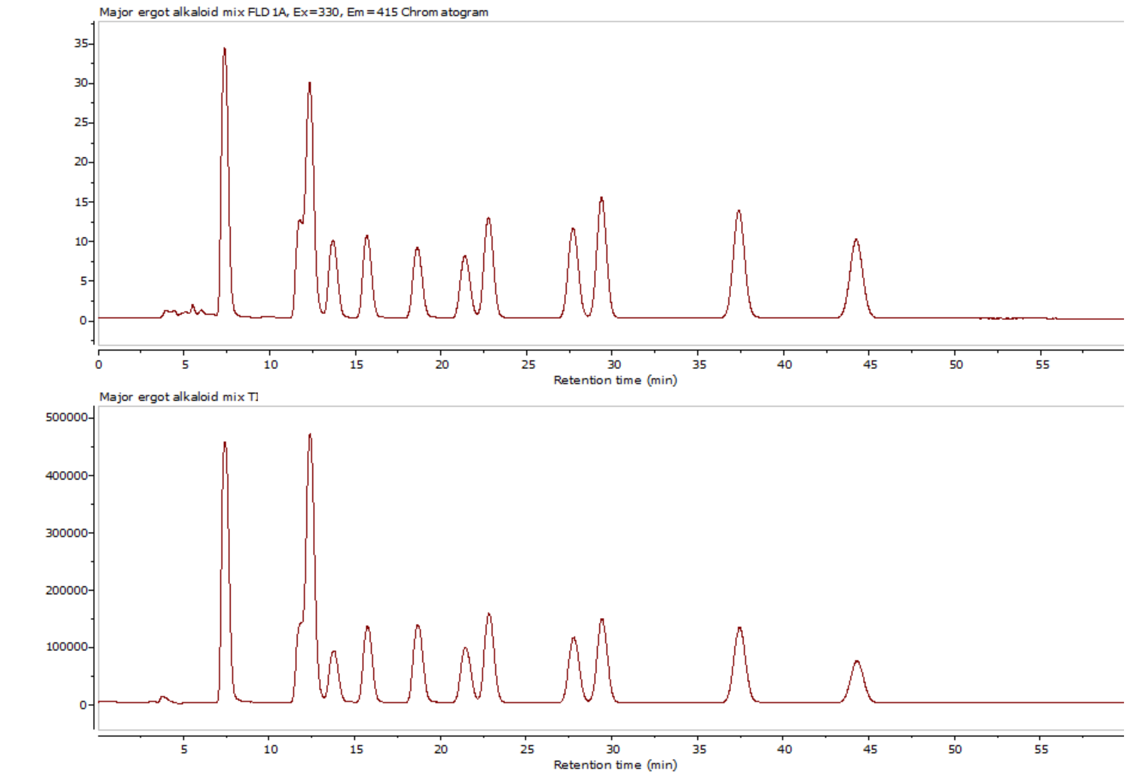


**Figure S1**: HPLC-FLD (top) and -MS (bottom) (SIM masses of the major ergot alkaloids (m/z $(M+H)^+$ = 326.4; 576.7; 548.6; 582.7; 610.7; and 562.1) and lysergic acid hydrazide (m/z $(M+H)^+$ = 283.1)) of the ergot alkaloid mix solution before hydrazinolysis.

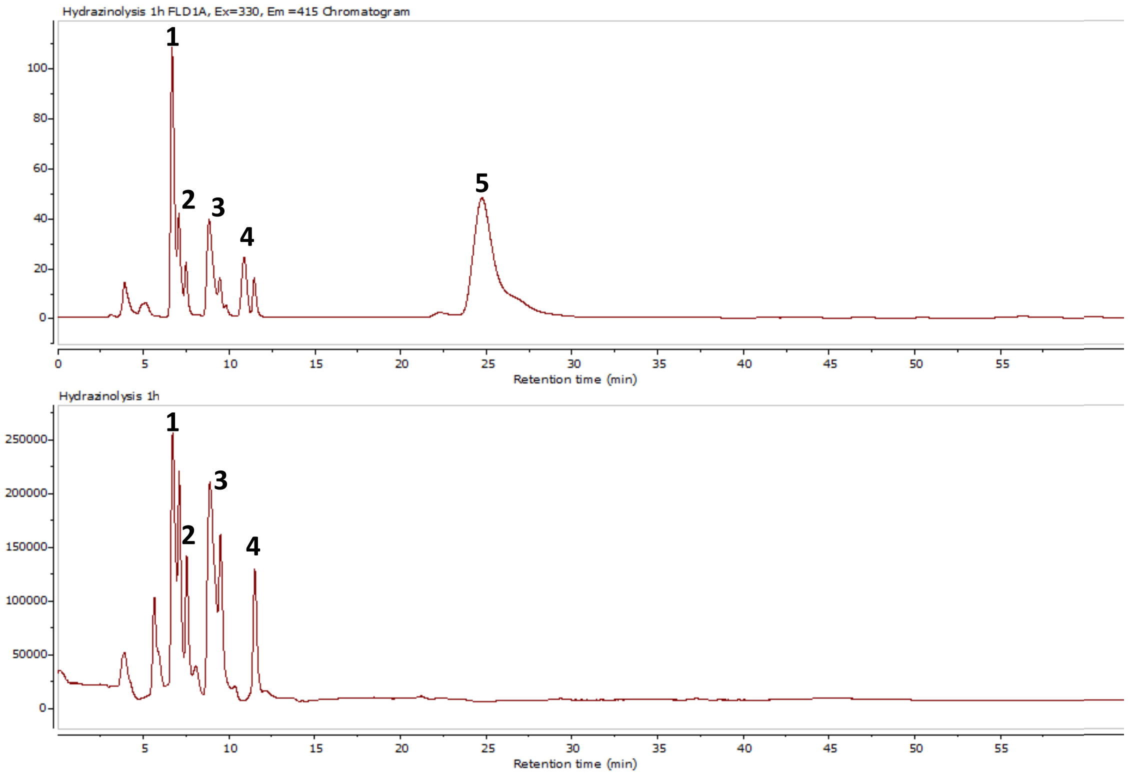

**Figure S9**: HPLC-FLD (top) and -MS (bottom) (SIM masses of the major ergot alkaloids (m/z $(M+H)^+$ = 326.4; 576.7; 548.6; 582.7; 610.7; and 562.1) and lysergic acid hydrazide (m/z $(M+H)^+$ = 283.1) of the ergot alkaloid mix solution after hydrazinolysis (same HPLC conditions as in S8). 1 and 3 are peaks of the two isomers of lysergic acid hydrazide. 2 is unreacted ergometrine and 4 unreacted ergometrinine. 5 is always present, when hydrazine hydrate is heated (also without ergot alkaloids).
